# Relations between the Arabic BFI-2-S and the PID-5-BF among Community Sample

**DOI:** 10.1192/j.eurpsy.2026.11473

**Published:** 2026-07-31

**Authors:** B. M. Alansari

**Affiliations:** Psychology, Kuwait University, kuwait, Kuwait

## Abstract

**Introduction:**

The BFI-2-S measures the Big Five personality factors including Extraversion, Agreeableness, Conscientiousness, Negative Emotionality, and Open-Mindedness. The PID-5- BF, however, is a brief measure of Personality Inventory for DSM-5, including Negative Affectivity, Detachment, Antagonism, Disinhibition, and Psychoticism.

**Objectives:**

The study examines the BFI-2-S in relation to a similar-length version of the PID-5-BF. The core purpose was to see how well the pathological traits of the PID-5-BF aligned with their theoretically corresponding domains in the established Five-Factor Model.

**Methods:**

The Arabic version of the PID-5-BF (25-item with 0–3 Likert-type scale) and the BFI-2-S (30-item with 1-5 Likert-type scale) scales were administered to 3900 Kuwait university undergraduates (1440 males mean age = 21.79±1.06 and 2460 females; mean age = 20.95±1.31).

**Results:**

Cronbach’s alphas ranged from 0.68 to 0.79 for the BFI-2-S Domains and 0.62 to 0.74 for the PID-5-BF Domains denoting moderate to acceptable internal consistency. The determined reciprocity pattern supports the theoretical expectations between the PID-5-BF and the BFI-2-S domains and confirms the relationship between normative and pathological personality. As expected, the PID-5-BF Negative Affectivity domain and the BFI-2-S Neuroticism (0.78), while the PID-5-BF Detachment, Antagonism, Disinhibition, and Psychoticism domains had negative relationships with the BFI-2-S Extraversion (–0.49), Agreeableness (–0.70), Conscientiousness (–0.76) and Openness (–0.85).

**Conclusions:**

This pattern indicates that most normal-range traits measured by the BFI-2-S inversely relate to maladaptive personality characteristics assessed by the PID-5-BF, supporting the theoretical continuity between adaptive and pathological personality functioning.

**Disclosure of Interest:**

None Declared

